# Clinical Patterns of Dyslipidemia in Patients With Initial-Treatment and Drug-Naïve Schizophrenia

**DOI:** 10.31083/AP46060

**Published:** 2025-08-26

**Authors:** Zhaopeng Kang, Lin Zhang, Tao Jiang, Guangya Zhang

**Affiliations:** ^1^Department of Psychiatry, Suzhou Guangji Hospital, The Affiliated Guangji Hospital of Soochow University, 215131 Suzhou, Jiangsu, China; ^2^Department of Psychiatry, Wuhan Mental Health Center, 430012 Wuhan, Hubei, China

**Keywords:** dyslipidemia, prevalence, schizophrenia, drug-naïve, initial treatment

## Abstract

**Background:**

Schizophrenia (SCZ) is a common, chronic, severe mental disorder that is often accompanied by dyslipidemia and linked to decreased life expectancy. The prevalence of dyslipidemia among initial-treatment and drug-naïve (ITDN) patients with SCZ and the correlates influencing its occurrence and severity were determined in this study.

**Methods:**

Demographic and clinical data including blood pressure, blood cell count, renal function, lipid profile, fasting glucose level, and thyroid function were collected from the 668 patients with ITDN SCZ included in this study. Psychopathology and illness severity were evaluated using the Positive and Negative Symptom Scale and the Clinical Global Impression Scale - Severity of Illness, respectively.

**Results:**

The prevalence of dyslipidemia was 33.53% (224/668) and the influencing factors included higher education attainment (B = 0.43, *p* = 0.018, odds ratio [OR] = 1.54) and elevated systolic blood pressure (SBP) (B = 0.04, *p* < 0.001, OR = 1.04), which were predictive factors. Conversely, having a spouse (B = –0.40, *p* = 0.026, OR = 0.67), higher red blood cell counts (B = –0.77, *p* < 0.001, OR = 0.47), and higher free tetraiodothyronine (FT_4_) levels (B = –0.06, *p* = 0.022, OR = 0.94) were protective factors. Specifically, elevated SBP (B = 0.01, t = 2.71, *p* = 0.007, 95% confidence interval [CI] = 0.00–0.01) predicted dyslipidemia severity, whereas higher FT_4_ levels (B = –0.02, t = –2.45, *p* = 0.015, 95% CI = –0.04–0.00) had a protective effect.

**Conclusions:**

Our study provides valuable insights into the clinical characteristics of dyslipidemia in ITDN SCZ patients. The identified factors influencing dyslipidemia occurrence and severity could serve as potential bioindicators for its prevention and intervention in clinical settings.

## Main Points

1. Dyslipidemia was observed in 33.53% of patients with initial-treatment and drug-naïve (ITDN) schizophrenia (SCZ).

2. Higher education level and higher systolic blood pressure (SBP) level were predictive factors for the development of dyslipidemia in patients with SCZ.

3. Elevated SBP was correlated with more severe forms of dyslipidemia in patients with SCZ.

## 1. Introduction

Schizophrenia (SCZ) is a chronic and severe mental disorder having a multifaceted etiology [1]. Increasing evidence highlights heightened symptoms that are predictive of secondary endocrine and metabolic disturbances in patients with SCZ [2]. Among these, dyslipidemia—a common metabolic condition—is commonly observed in this patient population [3, 4]. Cardiovascular disease associated with metabolic disorders such as dyslipidemia accounts for 17.4%–22.0% of premature deaths among patients with SCZ, surpassing the numbers associated with suicide as a cause of mortality [5, 6]. Dyslipidemia is strongly associated with adverse outcomes such as cognitive deficits, suicide attempts, and aggressive behaviors [7, 8, 9]. Therefore, understanding the clinical characteristics of dyslipidemia in patients with SCZ is pivotal in improving clinical management and addressing serious health challenges.

Several studies have focused on investigating the contribution of antipsychotics in metabolic dysregulation. Second-generation antipsychotics can induce metabolic disorders, including dyslipidemia [10, 11]. A study conducted in Singapore reported a striking overall prevalence of dyslipidemia of 62.7% in patients with SCZ exposed to antipsychotic drugs [12]. Increasing evidence suggests that subthreshold metabolic disorders may already be present during the premorbid phase of the disease and in individuals experiencing their first episode of psychosis [13]. Even in subjects in whom psychosis has been only predicted, the prevalence of metabolic syndrome has been found to be elevated and the levels of high-density lipoproteins (HDLs) to be lowered [14]. Evidence also suggests that SCZ may be associated with the altered synthesis of components such as cholesterol and fatty acids [15, 16]; furthermore, genome-wide association analyses have identified some loci where SCZ and dyslipidemia overlap [17, 18]. Overall, these findings highlight the presence of a natural genetic background that underlies the co-occurrence of dyslipidemia and SCZ.

To date, the effect of antipsychotic exposure on lipid levels in patients with SCZ has tended to be more favored. In contrast, relatively little attention has been paid to initial-treatment and drug-naïve (ITDN) patients [19, 20]. Only a few studies have reported the prevalence of diabetes mellitus, metabolic syndrome, and specific lipid component disorders in drug-naïve patients with SCZ, ignoring the overall prevalence of dyslipidemia [21, 22, 23]. The aim of this study was to investigate the prevalence of dyslipidemia and identify its influencing factors in a larger cohort of patients with ITDN SCZ to provide valuable guidance for the development of interventions targeted toward improving the long-term prognosis of this target population.

## 2. Patients and Methods

### 2.1 Subjects

The sample size was calculated using the following formula:

$$n=\frac{Z^{2}\,P\,\left(1-P\right)}{d^{2}}$$

where n represents the sample size, Z represents the Z-score corresponding to the desired confidence level (1.96 for 95% confidence interval (CI)), P is the estimated prevalence or proportion (set to 0.4 based on the prevalence of dyslipidemia in the general Chinese population), and d denotes the precision or allowable margin of error (fixed at 0.05). Using these parameters, the required sample size was estimated to be 369 participants.

**Fig. 1.**
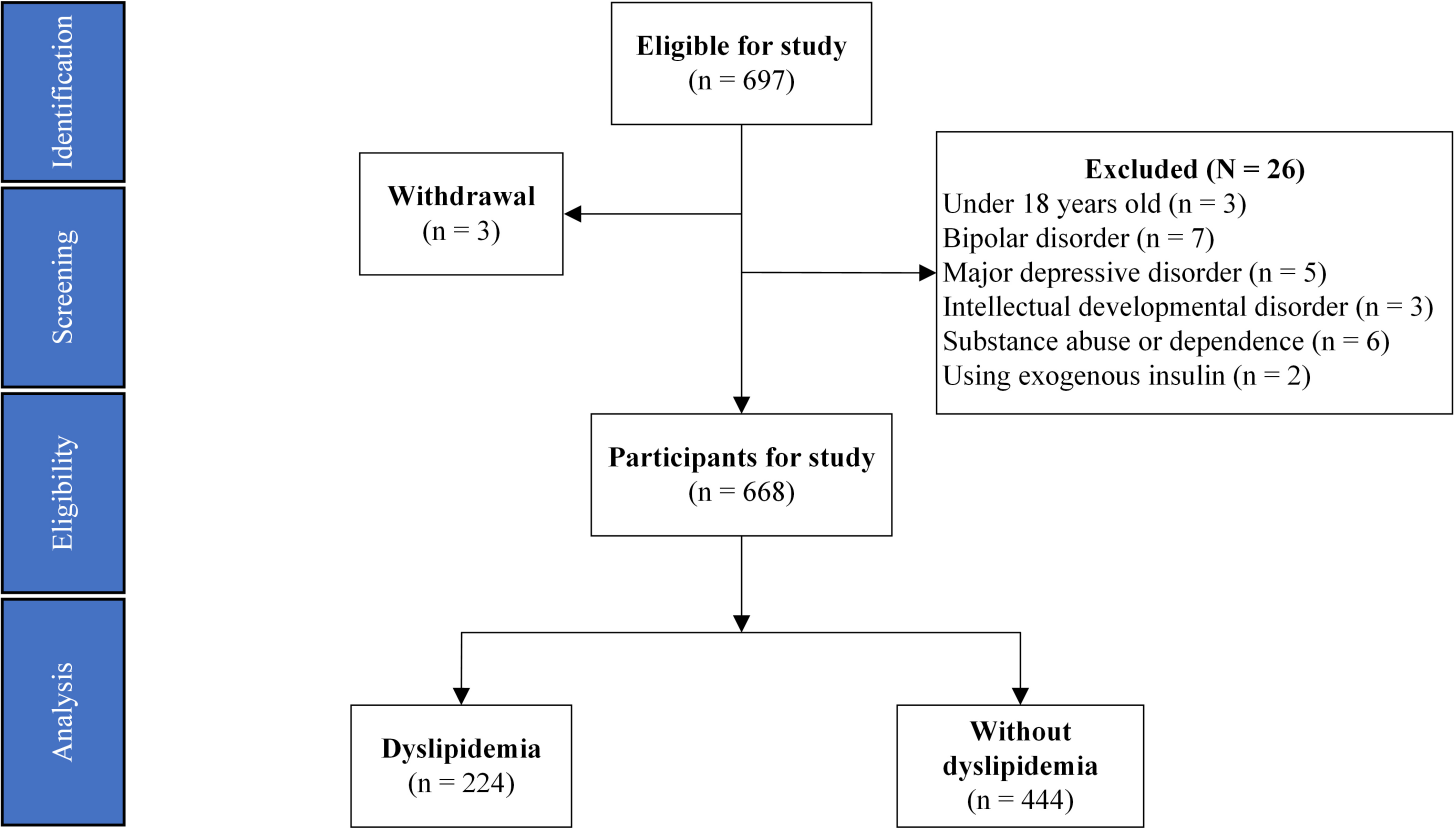
**Flowchart of study participants**.

A total of 668 ITDN patients diagnosed with SCZ who were hospitalized between February 2017 and June 2022 at Wuhan Mental Health Center were retrospectively included in this study. The flowchart and data source are detailed in Fig. 1.

The inclusion criteria were as follows:

1. Patients meeting the diagnostic criteria for SCZ as defined by the 10th revision of the International Classification of Diseases (ICD-10).

2. Drawing on the experience of Ma *et al*. [24], the Positive and Negative Symptom Scale (PANSS) score, which is used to assess the severity of psychopathology, scores at or above 60.

3. Age between 18 and 49 years, irrespective of gender.

4. Patients without prior exposure to antipsychotic medications before admission, although the use of benzodiazepines was permitted.

The exclusion criteria were as follows:

1. Individuals <18 years.

2. Patients with psychiatric conditions, including bipolar disorder, depression, intellectual disabilities, or substance use disorders.

3. Patients with significant physical illnesses (e.g., cardiopulmonary insufficiency, motor dysfunction, autoimmune diseases) or those recovering from surgery within 6 months.

4. Patients on lipid-lowering or glycemic-control medications.

Moreover, patients whose mental illnesses could not be definitively diagnosed as SCZ shortly after admission were monitored for up to 14 days. If alternative diagnoses could not be ruled out, they were withdrawn from the study.

Ethical approval for this study was provided by the Ethics Committee of the Wuhan Mental Health Center (ID: KY20170201.02).

### 2.2 Research Design

This cross-sectional study aimed to retrospectively determine the prevalence of dyslipidemia in the target population, and identify the factors associated with its development and severity.

Data collection: Demographic and clinical data were systematically recorded using a custom-designed Microsoft Excel spreadsheet. On the second day of admission, blood samples of each patient were drawn to assess biochemical markers, thyroid function, and other parameters, as detailed in Table 1, and extracted from the electronic medical records system.

**Table 1.** **The prevalence of dyslipidemia**.

Index	N	Prevalence
Overall	224	33.53%
Hyper-TC	36	5.39%
Hyper-TG	88	13.17%
Hyper-LDL-C	36	5.39%
Hypo-HDL-C	140	20.96%

TC, total cholesterol; TG, triglycerides; LDL-C, low density lipoprotein cholesterol; HDL-C, high density lipoprotein cholesterol.

Psychopathology assessment: The severity of psychiatric symptoms in patients was evaluated on the day of admission by four attending psychiatrists who had all undergone standardized training. Assessments were conducted using the PANSS and the Clinical Global Impression Scale - Severity of Illness (CGI-SI). The PANSS consists of 30 items, each of which is rated on a severity scale from 1 (nonexistent) to 7 (extreme). It was further subdivided into five dimensions (positive, negative, arousal, anxiety/depression, and cognition) for statistical analysis according to the method described by Kim *et al*. [25]. The CGI-SI scores range from 1 (healthy, not ill) to 7 (among the most severely ill).

Diagnosis of dyslipidemia: Based on the most recent official Chinese guidelines published in 2023, dyslipidemia was diagnosed if at least 1 of the following criteria was met [26]:

(1) Total cholesterol (TC) ≥5.2 mmol/L

(2) Triglycerides (TG) ≥1.7 mmol/L

(3) Low-density lipoprotein cholesterol (LDL-C) ≥3.4 mmol/L

(4) High-density lipoprotein cholesterol (HDL-C) <1.0 mmol/L

Derivation of the dyslipidemia score: Based on an earlier approach for scoring dyslipidemia [27], the first step involved calculating the reciprocal of HDL-C. Next, the four lipid indicators (TC, TG, LDL-C, and the reciprocal of HDL-C) were standardized to reflect the updated lipid profile. Next, principal component analysis with varimax rotation was applied to the standardized components, yielding principal components (PCs) that captured a substantial portion of the observed variability. In this analysis, PC1 accounted for 52.75% of the variance and PC2 for 30.47% [loading values for PC1 (PC2): TC 0.99 (–0.09), TG 0.47 (0.70), LDL-C 0.91 (0.03), and HDL-C –0.30 (0.85)]. The weights of the PC scores were determined based on the proportion of variance explained by PC1 and PC2. Lastly, individual weighted PC scores were aggregated to obtain the dyslipidemia score.

### 2.3 Statistical Analysis

Categorical variables are presented as counts, and continuous measurements as means with standard deviations or medians (p25, p75). The prevalence of dyslipidemia and its components was first determined. Next, the clinical parameters between subgroups were compared with and without dyslipidemia. The Shapiro–Wilk test was used to assess the normality of continuous variables, whereas *t*-tests, Mann–Whitney U-tests, and chi-square tests were used for variables with normal and non-normal distributions and for categorical variables. Next, a binary logistic regression model was developed to analyze the correlates of dyslipidemia, using dyslipidemia as the outcome and variables with significant differences in univariate analyses as predictors. Lastly, a multivariate linear regression model was used to identify the factors affecting the severity of dyslipidemia, with dyslipidemia score as the dependent variable and parameters from the previous step as predictors. Data were analyzed using IBM SPSS (version 27.0, IBM Corp., Armonk, NY, USA) and plotted using GraphPad Prism (version 8.4.3, GraphPad Software, Inc., La Jolla, CA, USA). All statistical tests were two-tailed with a significance level of *p* < 0.05.

## 3. Results

### 3.1 Prevalence of Dyslipidemia and Disorders in the Four Lipid Components

**Fig. 2.**
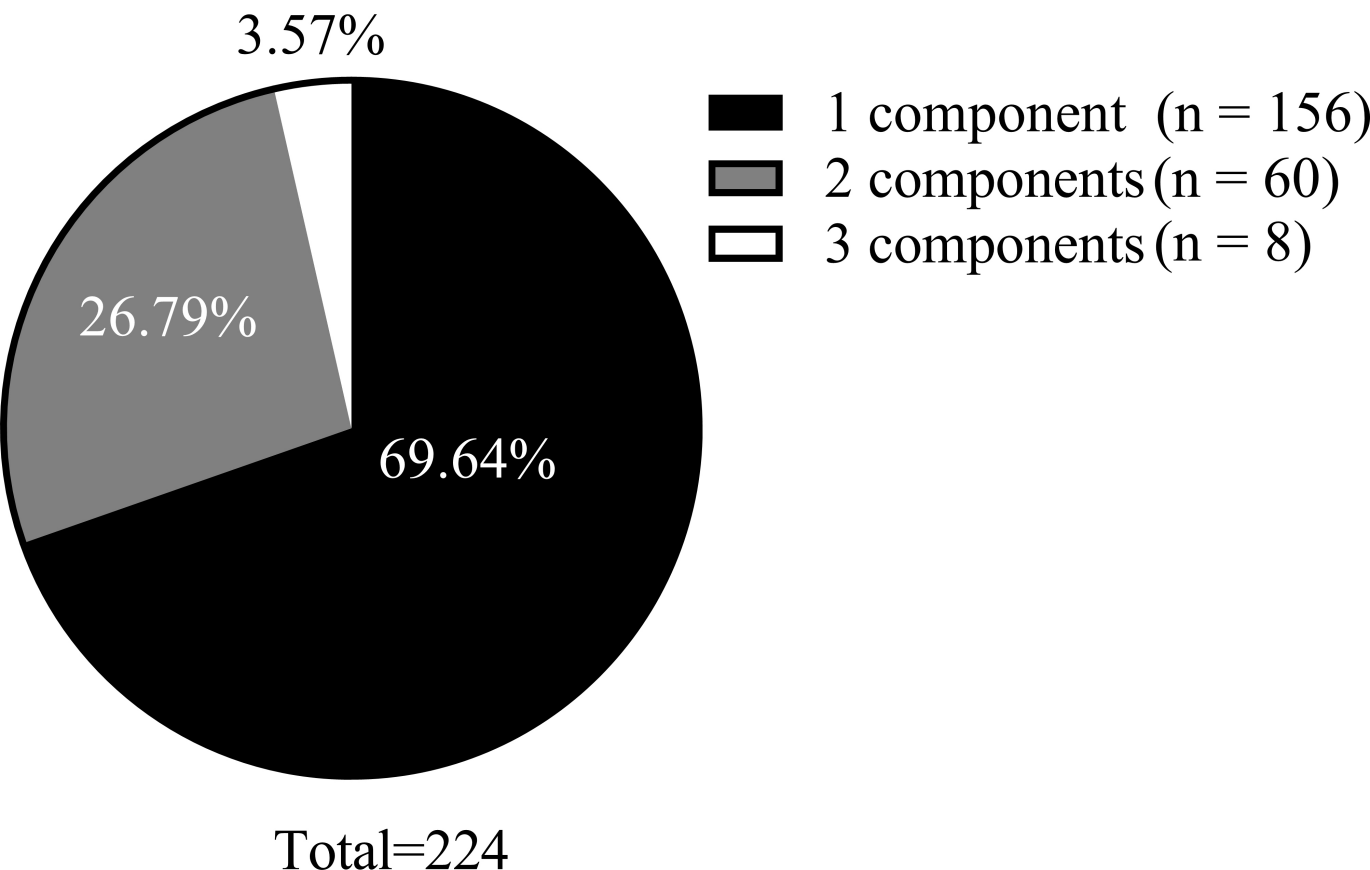
**Composition of the number of lipid component disorders**.

Of the 668 patients included in this study, 33.53% (224/668) had at least one type of dyslipidemia. The overall prevalence of the four distinct lipid components was as follows: 5.39% (36/668) for hyper-TC, 13.17% (88/668) for hyper-TG, 5.39% (36/668) for hyper-LDL-C, and 20.96% (140/668) for low hypo-HDL-C (Table 1). Within the dyslipidemia subgroup, 69.64% (156/224) of patients presented with only one dyslipidemia component, 26.79% (60/224) exhibited two components, and 3.57% (8/224) exhibited three dyslipidemia components. None of the patients had all four components. The components are shown in Fig. 2.

### 3.2 Differences in Clinical Parameters Between the Two Clinical Subgroups With and Without Dyslipidemia

Compared with the subgroup without dyslipidemia, the dyslipidemia subgroup had significantly lower scores for the onset age of psychiatric illness (Z = –2.35, *p* = 0.019), proportion with spouse ($\chi^{2}$ = 16.36, *p* < 0.001), proportion with low education attainment ($\chi^{2}$ = 11.41, *p* < 0.001), HDL-C levels (t = 13.41, *p* < 0.001), red blood cell (RBC) counts (t = 2.49, *p *= 0.013), creatinine (CRE) levels (t = 3.15, *p* = 0.002), and FT_4_ levels (t = 2.72, *p* = 0.007), whereas dyslipidemia scores (Z = –13.70, *p* < 0.001), TC levels (t = –3.39, *p* < 0.001), TG levels (t = –12.17, *p* < 0.001), LDL-C levels (t = –5.66, *p* < 0.001), and systolic blood pressure (SBP) (Z = –4.11, *p* < 0.001) and DBP (Z = –3.22, *p *= 0.001) were significantly higher (Table 2).

**Table 2.** **Sociodemographic and clinical parameters of included patients with and without dyslipidemia**.

Index		Total patients	Dyslipidemia	Non-dyslipidemia	t/Z/χ^2^	*p*-value
		(n = 668)	(n = 224)	(n = 444)		
Age - years		28 (23, 36)	28 (22, 33)	29 (23, 37)	–1.61	0.108
Disease duration - years		4 (2, 7)	3 (2, 6)	4 (1, 8)	–0.46	0.649
Onset age - years		24 (19, 29)	23 (19, 26)	24 (20, 29)	–2.35	0.019*
Gender - (n, %)					0.04	0.841
	Female	424, 63.47%	141, 65.98%	283, 63.74%		
	Male	244, 36.53%	83, 34.02%	161, 36.26%		
Marital status - (n, %)					16.36	<0.001*
	Spousal	312, 46.71%	80, 35.71%	232, 52.25%		
	Others	356, 53.29%	144, 64.29%	212, 47.75%		
Educational background - (n, %)					11.41	<0.001*
	Junior school and below	440, 65.87%	128, 57.14%	312, 70.27%		
	High school and above	228, 34.13%	96, 42.86%	132, 39.73%		
Dyslipidemia score		–0.08 (–0.28, 0.23)	0.26 (4 × 10^–⁢4^, 0.70)	–0.19 (–0.39, –3 × 10^–⁢3^)	–13.70	<0.001*
Lipid dimensions						
	TC - mmol/L	3.85 ± 0.72	4.00 ± 0.95	3.77 ± 0.55	–3.39	<0.001*
	TG - mmol/L	1.09 ± 0.55	1.49 ± 0.70	0.89 ± 0.31	–12.17	<0.001*
	LDL-C - mmol/L	2.18 ± 0.58	2.39 ± 0.74	2.08 ± 0.45	–5.66	<0.001*
	HDL-C - mmol/L	1.18 ± 0.23	1.02 ± 0.24	1.26 ± 0.18	13.41	<0.001*
WC - cm		78 (71.5, 84)	80 (70, 86)	78 (72, 83)	–1.74	0.082
SBP - mmHg		110 (110, 120)	114 (110, 120)	110 (100, 120)	–4.11	<0.001*
DBP - mmHg		75 (70, 80)	80 (70, 80)	70 (70, 80)	–3.22	0.001*
FBG - mmol/L		5.74 (5.56, 6.01)	5.72 (5.56, 6.01)	5.74 (5.56, 6.00)	–0.16	0.871
RBC - 10^12^/L		4.55 ± 0.47	4.49 ± 0.43	4.58 ± 0.49	2.49	0.013*
HGB - g/L		134 (123, 150)	133 (121, 148)	134 (124, 151)	–2.42	0.016*
WBC - 10^9^/L		6.91 ± 2.00	6.78 ± 1.84	6.98 ± 2.07	1.25	0.211
PLT - 10^9^/L		238 (203, 274)	242 (205, 276)	236 (202, 272)	–1.16	0.248
BUN - mmol/L		4.2 (3.2, 5.3)	4.3 (3.2, 5.3)	3.8 (3.1, 5.8)	–1.53	0.127
CRE - mmol/L		57.92 ± 12.56	55.59 ± 14.68	59.10 ± 11.18	3.15	0.002*
UA - mmol/L		413.19 ± 123.88	412.8 ± 106.61	413.39 ± 131.85	0.06	0.951
TSH - uIU/mL		1.80 (1.02, 2.29)	1.79 (0.95, 2.26)	1.8 (1.12, 2.31)	–1.35	0.178
FT_3_ - pmol/L		4.85 ± 0.69	4.81 ± 0.69	4.87 ± 0.68	0.93	0.354
FT_4_ - pmol/L		16.94 ± 3.15	16.51 ± 2.77	17.17 ± 3.31	2.72	0.007*
CGI-SI		5 (5, 6)	5 (5, 6)	5 (5, 6)	–0.91	0.364
PANSS		88.90 ± 11.43	89.20 ± 13.63	88.76 ± 10.15	–0.43	0.670
	Positive factor	15.90 ± 3.51	16.04 ± 3.77	15.83 ± 3.37	–0.69	0.489
	Negative factor	28.23 ± 6.22	28.05 ± 6.81	28.32 ± 5.91	0.51	0.608
	Excitement factor	12.71 ± 4.44	13.14 ± 5.01	12.50 ± 4.11	–1.67	0.095
	Anxiety/Depression factor	15.43 ± 4.50	15.21 ± 5.22	15.53 ± 4.09	0.79	0.427
	Cognitive factor	16.48 ± 4.41	16.70 ± 4.32	16.37 ± 4.45	–0.91	0.366

WC, waist circumference; SBP, systolic blood pressure; DBP, diastolic blood pressure; FBG, fasting blood glucose; RBC, red blood cell; HGB, hemoglobin; WBC, white blood cell; PLT, platelet; BUN, blood urea nitrogen; CRE, blood creatinine; UA, blood uric acid; TSH, thyroid stimulating hormone; FT_3_, free triiodothyronine; FT_4_, free tetraiodothyronine; CGI-SI, Clinical Global Impression Scale - Severity of Illness; PANSS, Positive and Negative Syndrome Scale. *p < 0.05.

### 3.3 Correlates for Dyslipidemia: Based on a Binary Logistic Regression Model

A binary logistic regression model (Backward: Wald) was used with dyslipidemia as the dependent variable to investigate the correlates for dyslipidemia. The independent variables were those found to differ significantly in the prior univariate analysis, excluding the four lipid components and the dyslipidemia score (Table 3). The factors associated with dyslipidemia were identified, specifically, higher education attainment (B = 0.43, *p* = 0.018, odds ratio (OR) = 1.54) and higher SBP (B = 0.04, *p* < 0.001, OR = 1.04) as predictive factors; having a spouse (B = –0.40, *p* = 0.026, OR = 0.67) and higher RBC levels (B = –0.77, *p* < 0.001, OR = 0.47) and FT_4_ (B = –0.06, *p* = 0.022, OR = 0.94) as protective measures.

**Table 3.** **Factors influencing dyslipidemia in all included patients: a binary logistic regression model**.

	Coefficients	Std. error	Wald	*p *value	95% CI for OR		
	B				OR	Lower	Upper
Constant	–0.33	1.12	0.08	0.772	0.72		
Educational background (high *vs.* low)	0.43	0.18	5.62	0.018*	1.54	1.08	2.20
Marital status (spousal *vs.* others)	–0.40	0.18	4.95	0.026*	0.67	0.47	0.95
SBP - mmHg	0.04	0.01	24.47	<0.001*	1.04	1.02	1.05
RBC - 10^12^/L	–0.77	0.20	15.00	<0.001*	0.47	0.32	0.69
FT_4_ - pmol/L	–0.06	0.03	5.26	0.022*	0.94	0.89	0.99

*p < 0.05. CI, confidence interval; OR, odds ratio.

### 3.4 Correlates Associated With Dyslipidemia Scores: Based on a Multiple Linear Regression Model

A multiple linear regression model (Backward method) was used to identify the factors influencing dyslipidemia scores in the subgroup of individuals diagnosed with dyslipidemia (Table 4). The dyslipidemia score was set as the dependent variable, and the clinical parameters previously identified as significant in the binary logistic regression analysis served as independent variables. Our findings indicated that higher FT_4_ level (B = –0.02, t = –2.45, *p* = 0.015, 95% CI = –0.04–0.00) was a protective measure for dyslipidemia score and that higher SBP (B = 0.01, t = 2.71, *p* = 0.007, 95% CI = 0.00–0.01) was a predictive factor.

**Table 4.** **Correlates affecting dyslipidemia scores: a multiple linear regression model**.

	Coefficients	Std. error	*t*	*p-*value	95% CI for B	
	B				Lower	Upper
Constant	0.06	0.28	0.22	0.825	–0.49	0.61
SBP - mmHg	0.01	0.00	2.71	0.007*	0.00	0.01
FT_4_ - pmol/L	–0.02	0.01	–2.45	0.015*	–0.04	0.00

*p < 0.05.

## 4. Discussion

This study reports the overall prevalence of dyslipidemia in patients with ITDN SCZ, details the prevalence of specific lipid-component abnormalities, and identifies the correlates influencing the occurrence and severity of dyslipidemia. Collectively, these findings offer important insights for guiding prevention and intervention strategies in clinical practice.

Metabolic disturbances in individuals with SCZ may be endogenous in nature [28]. Several studies have reported dyslipidemia in patients with SCZ that pre-dates exposure to antipsychotics [13, 29, 30]. The current study reports an overall prevalence of dyslipidemia of 33.53% in patients with ITDN SCZ as well as the prevalence of the four lipid components to be 5.39% for hyper-TC, 13.17% for hyper-TG, 5.39% for hyper-LDL-C, and 20.96% for hypo-HDL-C. Our findings are aligned with a report published on the prevalence of dyslipidemia in the ITDN population with SCZ in northern China [30]. Another research group has determined the prevalence and factors affecting metabolic syndrome in patients with first-episode SCZ in northern China. Their findings revealed the prevalence to be 16.5% for hyper-TC, 30.5% for hyper-TG, 21.4% for hyper-LDL-C, and 43.1% for hypo-HDL-C [31]. These reported prevalence rates were higher than ours. Upon comparison, it was found that the study defined hyper-TC, hyper-LDL-C, and hypo-HDL-C more loosely and included patients with a higher mean age, which may be the main reason responsible for their higher prevalence rates. After pooling the results from a large sample meta-analysis, the results were found to be similar to those reported in our study, i.e., the prevalence of hypertriglyceridemia in patients with drug-naïve SCZ was 16.9% and the rate of hypo-HDL-C was 20.4% [32]. Overall, our findings related to the prevalence of dyslipidemia in patients with ITDN SCZ align more closely with those from previous studies. Most notably, our results provide robust evidence that supports the coexistence of dyslipidemia in patients with ITDN SCZ prior to the initiation of antipsychotic therapy.

Next, the determinants of dyslipidemia development in the enrolled subjects were identified. Our analysis revealed that high education levels and high SBP were associated with an increased prediction of dyslipidemia. Conversely, being married, having higher RBC counts, and having higher CRE and FT_4_ levels were determined to be protective factors against dyslipidemia. Several studies from Europe and the Americas are more inclined to suggest that high levels of education help populations become immune to metabolic disorders [33, 34, 35]. This finding is contradicts the results of our analysis, wherein highly educated people in China were found to be more likely to be employed in jobs associated with sedentary lifestyles [36, 37]. Elevated blood lipid levels and blood pressure are the two main predictive factors of cardiovascular disease that are often known to co-occur. The National Health and Nutrition Examination Surveys have found that approximately 60.7%–64.3% of patients with hypertension also have hypercholesterolemia [38]. Many studies have shown a linear correlation between blood pressure and serum cholesterol levels [39, 40, 41]. This evidence provides equally robust support for our report. Marital status is believed to function as an indirect adjustment of metabolic parameters [42]. A cross-sectional study has confirmed that improved marital relationships were associated with lower LDL-C and TC levels [43]. Collectively, our findings suggest that metabolic parameters, including lipid profiles, may receive more attention and intervention in patients who have spouses. FT_4_ was another protective measure for dyslipidemia that was identified in our study. FT_4_ levels were elevated in patients with SCZ than in the healthy controls [44], and subsequent prospective studies and Mendelian randomization studies have further established the protective effect of reasonably high FT_4_ levels against dyslipidemia [45, 46]. Therefore, it is reasonable to deduce that maintaining a certain FT_4_ level may help correct dyslipidemia in patients.

To assess the severity of dyslipidemia, the continuous variables for dyslipidemia were transformed, and dyslipidemia scores were calculated. Subsequently, higher SBP was identified as a predictive factor for dyslipidemia severity, and higher FT_4_ level was determined to be a protective measure. However, only a few studies have evaluated the severity of dyslipidemia. Overall, blood pressure is strongly correlated with lipid levels, especially high TG and low HDL-C levels [47]. Studies from Nigeria and Bangladesh have reported a significant increase in plasma TC, LDL-C, and TG levels with an increase in blood pressure [48, 49], with a strong negative correlation with HDL-C levels [50]. Collectively, it is not difficult to deduce that SBP could serve as a conclusion of the severity of dyslipidemia. With respect to the protective measures for the degree of dyslipidemia, a two-sample bidirectional Mendelian randomization study found that FT_4_ levels could significantly reduce the prediction of metabolic syndrome, which also includes a reduction in the predictors of the components of metabolic syndrome including high waist circumference and low HDL-C levels [51, 52]. Furthermore, high FT_4_ levels are negatively correlated with TG levels than low and intermediate levels [52, 53]. This evidence parallels our findings to a certain extent, implying the benefits of FT_4_ in reducing the severity of dyslipidemia.

Our study has some limitations. First, due to its cross-sectional design, it was not possible to establish a causal relationship between access to predictive factors identified in the study and the development of dyslipidemia. Second, our sample population included patients who were not exclusively first-episode individuals. While none of the patients had a history of antipsychotic exposure, some had undergone a more prolonged disease course, which introduced additional confounding factors. Third, age is a crucial determinant of dyslipidemia; however, age stratification was not considered during statistical analysis because of the limited sample size. Fourth, dietary habits significantly influence dyslipidemia; however, the current study did not thoroughly examine the dietary profiles of patients enrolled in this study. These factors may have collectively compromised the statistical efficacy of age as a factor in our data analysis. In future studies, more rigorous prospective designs and larger sample sizes would be considered to address these limitations.

## 5. Conclusions

Our study provides valuable insights into the clinical characteristics of dyslipidemia in patients with ITDN SCZ. The factors influencing the occurrence and severity of dyslipidemia that were identified in this study could likely be used as potential bioindicators in a clinical setting for the prevention and intervention of dyslipidemia in SCZ populations.

## Availability of Data and Materials

The datasets used and/or analyzed during the current study are available from the corresponding author upon reasonable request.
